# Fear of COVID-19 and Prevention Behaviors: Cross-Lagged Panel Analysis

**DOI:** 10.2196/35730

**Published:** 2022-11-17

**Authors:** Katherine M Anderson, Jamila K Stockman

**Affiliations:** 1 Department of Medicine School of Medicine University of California San Diego La Jolla, CA United States; 2 Department of Behavioral, Social, and Health Education Sciences Rollins School of Public Health Emory University Atlanta, GA United States

**Keywords:** fear appeals, structural equation modelling, cross-lagged model, prevention behavior, COVID-19, fear, women, behavior, change, health, physical distance, relationships, pandemic, research, association, prevention, experience, panel, interest, public, distancing

## Abstract

**Background:**

The ongoing COVID-19 pandemic has brought forth conversations about effective behavior change models for increasing prevention behavior, ranging from wearing masks in public to physical distancing. Among the considered behavior change techniques is the use of fear appeals, through which a negative possible outcome is emphasized to invoke fear, which in turn may promote prevention behaviors to counter the likelihood of the negative outcome. Although fear is hypothesized as health promoting in some theories of health behavior, little research has rigorously assessed the relationship.

**Objective:**

In our exploratory analyses, we aim to examine the association, including directionality of the association between fear of COVID-19 and COVID-19 prevention behaviors across 2 time points during the early COVID-19 pandemic among a sample of US women.

**Methods:**

The COPE study, a web-based survey of US women’s COVID-19 experiences, was deployed in May-June 2020 (time 1) with follow-up in December 2020-January 2021 (time 2; n=200). Demographic characteristics as well as fear of COVID-19 and COVID-19 prevention behaviors (eg, staying home except for essential activities, physical distancing in public, and masking in public) were measured. Descriptive and bivariate analyses were used to characterize COVID-19 prevention behaviors and fear of COVID-19 among participants. Cross-lagged panel analysis, a type of structural equation modeling that assesses directionality of temporal associations, was used to understand relationships, if any, between variables of interest.

**Results:**

We found cross-sectional associations between fear of COVID-19 and staying home and physical distancing, as well as temporal associations between fear at time 1 and time 2 and prevention behaviors at time 1 and time 2. However, results of the cross-lagged panel analysis indicated no cross-lagged temporal relationships between fear of COVID-19 and COVID-19 prevention behaviors 6 months apart.

**Conclusions:**

Fear of COVID-19 did not appear to predict COVID-19 prevention behaviors 6 months after initial measurements among the sample of women recruited for our study. Future research should rigorously test these associations longitudinally, and alternative methods of public health prevention promotion should be considered.

## Introduction

Amid the ongoing COVID-19 pandemic, behavioral prevention is vital to reduce viral transmission. Uptake of prevention behaviors, including masking in public, social distancing, and staying home except for essential activities [1], has been variable [2,3], underscoring the need for identification of mechanisms of behavior change to encourage uptake of behaviors appropriate for the current level of risk and dominant variants. Concerningly, it has been suggested that those who have previously been infected with COVID-19 are even less likely to use prevention behaviors [4], despite risk of reinfection and continued evolution of variants. As a mechanism for behavior change, fear appeals have long been used in public health [5] and justified with behavioral theory constructs of perceived risk or perceived severity, such as within the Health Belief Model [6-8]. Results are mixed on the effectiveness [9] and ethics [10] of fear appeals. Significant literature has emerged on the role of fear of COVID-19 [2,11,12], though somewhat less on fear and its association with prevention behavior [3,8]. The available literature has identified positive cross-sectional associations between anxiety or fear of infection related to COVID-19 and willingness to vaccinate [13], with odds of vaccine hesitancy approximately 5.5 times greater among those with no fear of COVID-19 compared to those with a great extent of fear [14]. Fear has also been demonstrated to mediate the relationship between COVID-19 exposure and intent to be vaccinated [15] and the relationship between COVID-19 information seeking and prevention behavior performance [16]. Further, COVID-19 fear is predictive of behavioral intention to perform prevention behaviors [17,18], and fear of contamination is predictive of obsessive-compulsive hand washing [19]. In contrast to this evidence, longitudinal studies have found that as the pandemic progressed, fear diminished over time while prevention behaviors increased, even as uncertainty related to the pandemic remained stable [20].

Arguments for fear appeals are based on assumed directionality from fear of an outcome to behavior preventing that outcome. This does not take into account the potential of promotion and use of prevention behaviors increasing anxiety or fear related to COVID-19 nor does it consider competing fear, such as the negative impacts of social isolation resulting from prevention behaviors [21,22]. However, most studies of fear appeals do not thoroughly assess directionality or further interactions such as mediation or moderation [23,24], with few exceptions [25]. As such, this analysis attempts to answer the following research question: what is the directionality of the relationship between fear of COVID-19 and practice of mask wearing, physical distancing in public, and staying home except for essential activities across 2 time points 6 months apart among adult US women enrolled in an internet-based study?

## Methods

### Ethical Considerations

All procedures were approved by the University of California San Diego’s (project 200663) institutional review board. Participants provided documented informed consent prior to completing surveys at each time point.

### Procedures

Participants were recruited for The COPE Study baseline survey from May to June 2020 (time 1 [t_1_]), using the Facebook advertising algorithm. Women aged ≥18 years were targeted for advertisements on Facebook (83.1%) and other non-Facebook–owned websites on which the program anticipates reaching the demographic of interest (Facebook Audience Network: 16.5%). The aim of The COPE Study was to understand US adult women’s experiences with COVID-19, service access, and violence during the first months of the COVID-19 pandemic. Of 682 potential participants, 633 (92.8%) provided consent and responded to eligibility questions. Eligible participants were ≥18 years of age, self-identified as women, lived in the United States, and could speak and understand English; of 626 eligible participants, 491 (78%) completed the internet-based survey at t_1_. For the follow-up survey, administered between December 2020 and January 2021 (time 2 [t_2_]), we conducted 2-stage stamping. In stage 1, all non-White participants were purposively sampled for overrepresentation of racial or ethnic minorities. Due to the underrepresentation of racial and ethnic minority individuals in research, this tactic helped to bolster the diversity of our sample. In stage 2, we used simple random sampling of the remaining participants, not inclusive of nonresponders from the first stage of sampling, until achieving a sample size of N=333, due to funding constraints. Each participant was emailed an invitation to participate in the follow-up survey at an email address provided at t_1_, along with permission to recontact them. All data were collected using REDCap [26]. Participants were compensated with Amazon e-gift cards (t_1_: US $20; t_2_: US $15).

### Participants

Of 333 participants invited, 296 completed the survey at t_2_ (88.9%), and 200 (60%) passed response validity tests. Validity testing, which was only performed at t_2_, took the form of an attention check, wherein participants were instructed to select a specific response to a question at 2 independent points in the survey. Of the remaining cases, 4/200 (2%) had missing data. Not accounting for data removed due to failed validity testing, 200/237 (84%) of invited participants completed the survey (200 of 237 participants not known failed validity checks). Median age of participants was 33 (IQR 18-69); 118/200 (59%) were White; 27/200 (13.5%) were Black; 16/200 (8%) were of Asian descent; and 9/200 (4.5%) identified as being multiracial; 27/200 (13.5%) were Hispanic or Latinx. Most participants had completed college (n=74/200, 37%) or had at least some graduate school education (n=62/200, 31%); 123/200 (62.5%) were employed, with 47/200 (23.5%) self-identified as essential workers.

### Measures

Four variables of interest were considered. Fear of COVID-19 was measured using the 7-item Fear of COVID-19 Scale [27]. Items included being afraid of dying from COVID-19 and experiencing physical symptoms of fear. Response options were on a 5-point Likert scale from “strongly disagree” to “strongly agree.” Responses were summed, with scores ranging from 7 to 35 (Cronbach α=.897 at t_1_; Cronbach α=.904 at t_2_). The following 3 prevention behaviors were measured: (1) staying home except for essential activities, (2) physical distancing of 6 feet from nonhousehold members, and (3) using a face mask in public. Participants were asked, “which of the following prevention behaviors have you been using?” with response options of “yes” or “no” for each.

### Statistical Analyses

Descriptive statistics were calculated, and 2-tailed independent samples *t* tests were run using IBM SPSS (version 24; IBM Corp). Given multiple comparisons, a Bonferroni adjustment and significance was set at *P*=.008 for *t* tests. Cross-lagged panel analysis [28] was conducted in Mplus (version 8) [29]. All responses were retained through maximum likelihood estimation with robust standard errors, using the weighted least squares mean and variance adjusted estimator in MPlus [30]; significance was set at *P*<.05. In total, 3 relationships of interest were assessed over 2 time points, as follows: (1) practice of staying home except for essential activities during the COVID-19 pandemic and fear of COVID-19, (2) practice of physical distancing in public during the COVID-10 pandemic and fear of COVID-19, and (3) practice of wearing a mask in public during the COVID-19 pandemic and fear of COVID-19. Age, formal educational attainment, and parental status (t_1_), as well as essential worker status (t_2_) were entered as time-invariant covariates in adjusted models. As a saturated cross-lagged model with 2 time points, goodness-of-fit indices are not used for model interpretation [31].

## Results

Demographic characteristics of the sample and distribution of the predictor and outcome variables are presented in Table 1 and Table 2; bivariate results are presented in Table 3. Prevention behaviors were practiced by most participants across time points, though the proportion of ‘staying home except for essential activities’ declined from t_1_ to t_2_ (93.5% to 78.5%), and the proportion of ‘wearing masks in public’ increased (83.5% to 95.5%), whereas the proportion of ‘physically distancing in public’ remained approximately the same (90% at t_1_ and 89% at t_2_). Fear of COVID-19 at t_1_ and t_2_ were not significantly different (mean difference 0.460; *P*=.25, not depicted) and were significantly correlated (*r*=0.662; *P*<.001; Table 4). Mean fear of COVID-19 was higher among women who stayed home except for essential activities at t_2_; however, upon correction for multiple comparisons, it was not significant (20.87 vs 19.44; *P*=.047), and it was significantly higher among women who maintained physical distance of 6 feet in public at both t_1_ (21.23 vs 17.0; *P*=.008) and t_2_ (20.82 vs 16.10; *P*=.005). Across other prevention behaviors and time points, fear and prevention behaviors were not statistically significantly associated.

**Figure 1:**
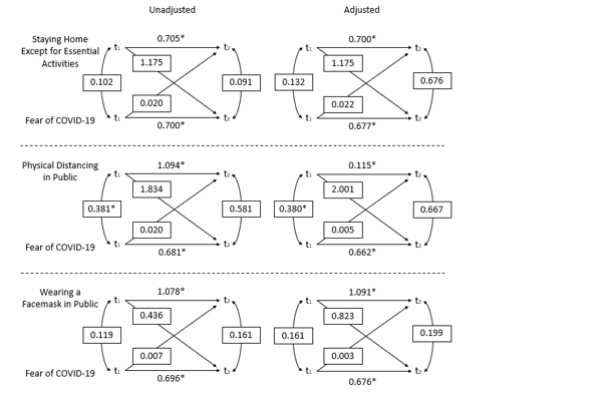
Cross-lagged panel models of prevention behavior and fear of COVID-19 among a sample of US adult women. *Denotes statistical significance at a value of .05. Adjusted models are adjusted for age, education, parental status, and essential worker status. t_1_: time 1. t_2_: time 2.

Beta estimates for each cross-lagged panel model are presented and depicted in Figure 1, and beta estimates and correlations as well as significance of associations at a level of *P*<.05 are presented in Table 4. Results of the cross-lagged models indicate that fear of COVID-19 (t_1_) does not predict practicing prevention behaviors 6 months later (t_2_), including staying home except for essential activities (adjusted model: β=.022; *P*=.11), physically distancing in public (adjusted model: β=.005; *P*=.74), or wearing a mask in public (adjusted model: β=.003; *P*=.86). Relatedly, practicing of prevention behaviors (t_1_) did not predict fear of COVID-19 6 months later (t_2_) for staying home except for essential activities (adjusted model: β=1.577; *P*=.31), physically distancing in public (adjusted model: β=2.001; *P*=.08), or wearing a mask in public (adjusted model: β=.823; *P*=.31). Fear of COVID-19 was strongly and significantly associated at t_1_ and t_2_ across all models (*P*<.001 for all), and prevention behavior at t_1_ was significantly associated with prevention behavior at t_2_ across all models (staying home: adjusted *P*=.02; distancing in public and wearing a mask in public: adjusted *P*<.001). Finally, physical distancing in public at t_1_ was statistically significantly associated with fear of COVID-19 at t_1_ (adjusted model: β=.380; *P*=.008).

**Table 1:** Demographic characteristics among a sample of US adult women (N=200).

Variables			Values
Age (years), mean (SD)			34.89 (11.1)
**Race, N (%)^a^**			
	White	118 (59)	
	Black	27 (13.5)	
	Asian	16 (8)	
	American Indian and Alaska Native	5 (2.5)	
	Multiple races	9 (4.5)	
	Middle Eastern and North African	3 (1.5)	
	Pacific Islander	0 (0)	
Ethnicity (Hispanic or Latinx), n (%)			27 (13.5)
**Education, n (%)**			
	High school diploma, GED^b^, or less	28 (14)	
	Some college, or some (or completed) trade or vocational school	34 (17)	
	Completed college	74 (37)	
	Some (or completed) graduate school	62 (31)	
Employed, n (%)^c^			125 (62.5)
In a relationship, n (%)			128 (64)
Parent to children of any age, n (%)			100 (50)
Essential worker, n (%)			47 (23.5)

^a^Participants selected all applicable races; some participants did not provide a race, identifying only as Hispanic or Latinx.

^b^GED: Graduate Educational Development.

^b^Includes full-time and part-time employees as well as self-employed.

**Table 2:** Variables of interest among a sample of US adult women (N=200).

Variables of interest	Time 1 (baseline)	Time 2 (6-month follow-up)
Staying home, n (%)	187 (93.5)	157 (78.5)
Physical distancing, n (%)	180 (90)	178 (89)
Masking in public, n (%)	167 (83.5)	191 (95.5)
Fear of COVID-19, mean (SD)	20.81 (6.76)	20.35 (7.11)

**Table 3:** Bivariate analyses of prevention behavior and fear of COVID-19 among a sample of US adult women (N=200). Italicized *P* values are significant.

Prevention behaviors				Fear of COVID-19						
				Time 1 (baseline), mean (SD)		*P* value		Time 2 (6-month follow-up), mean (SD)		*P* value
**Staying home**										
	**Time 1**									
		Yes	20.92 (6.7)		0.39		20.5 (7.06)		0.25	
		No	19.23 (7.64)				18.15 (7.86)			
	**Time 2**									
		Yes	21.18 (6.66)		.13		20.87 (6.97)		.05	
		No	19.44 (7.00)				19.44 (7.39)			
**Physical distancing**										
	**Time 1**									
		Yes	21.23 (7.48)		*.008*		20.82 (6.93)		*.005*	
		No	17.0 (7.97)				16.10 (7.48)			
	**Time 2**									
		Yes	20.92 (6.75)		.53		20.58 (7.06)		.19	
		No	19.95 (6.93)				18.45 (7.45)			
**Masking in public**										
	**Time 1**									
		Yes	20.95 (6.51)		.51		20.52 (6.77)		.52	
		No	20.09 (7.97)				19.48 (8.72)			
	**Time 2**									
		Yes	20.86 (6.59)		.64		20.40 (7.08)		.63	
		No	19.78 (10.07)				19.22 (8.18)			

**Table 4:** Estimated betas and correlations for cross-lagged models of prevention behavior and fear of COVID-19 among a sample of US adult women (N=200). Italicized values are significant.

Regression models		Unadjusted			Adjusted^a^	
		Estimate	*P* value	Estimate		*P* value
**Model 1**						
	Staying home (t_1_^b^ to t_2_^c^)	*.705*	*.05*	*.700*		*.02*
	Fear (t_1_ to t_2_)	*.700*	*<.001*	*.677*		*<.001*
	Staying home (t_1_) to fear (t_2_)	1.175	.54	1.577		.317
	Fear (t_1_) to staying home (t_2_)	.020	.15	.022		.13
	Staying home (t_1_) with fear (t_1_)	.102	.36	.132		.22
	Staying home (t_2_) with fear (t_2_)	.646	.18	.676		.17
**Model 2**						
	Distancing (t_1_ to t_2_)	*1.094*	*<.001*	*1.15*		*<.001*
	Fear (t_1_ to t_2_)	*.681*	*<.001*	*.662*		*<.001*
	Distancing (t_1_) to fear (t_2_)	1.834	.68	2.001		.08
	Fear (t_1_) to distancing (t_2_)	.002	.91	.005		.74
	Distancing (t_1_) with fear (t_1_)	*.381*	*.01*	*.380*		*.008*
	Distancing (t_2_ with fear (t_2_)	.581	.31	.677		.25
**Model 3**						
	Masking (t_1_ to t_2_)	*1.078*	*<.001*	*1.091*		*<.001*
	Fear (t_1_ to t_2_)	*.696*	*<.001*	*.676*		*<.001*
	Masking (t_1_) to fear (t_2_)	.436	.60	.823		.31
	Fear (t_1_) to masking (t_2_)	.007	.67	.003		.86
	Masking (t_1_) with fear (t_1_)	.119	.45	.161		.30
	Masking (t_2_) with fear (t_2_)	.161	.81	.199		.75

^a^Models are adjusted for age, formal educational attainment, and parental status (time 1) as well as essential worker status (time 2).

^b^t_1_: time 1 (baseline).

^c^t_2_: time 2 (6-month follow-up).

## Discussion

### Principal Findings

Findings from this exploratory analysis indicate that although fear of COVID-19 and practice of prevention behaviors may be self-predictive over 6 months, they are not cross-predictive among the cohort of US women enrolled in The COPE Study. In bivariate analyses, significant associations were found between fear and staying home except for essential activities at t_1_ and fear and physical distancing in public at both time points; however, further exploration of these relationships using cross-lagged panel analysis suggests these may not be temporal associations.

### Comparison With Prior Work

Previous literature has examined the use of fear appeals in behavioral health interventions, largely with mixed or inconclusive findings. Conflicting meta-analyses have suggested that fear appeals are not sufficiently effective [6] and that ‘strong’ fear appeals are very effective [9]. In the context of COVID-19, studies have found that fear is cross-sectionally associated with compliance with behavioral prevention [23] as well as willingness and intention to be vaccinated [13,14], and it is longitudinally associated with intention to perform prevention behaviors [17,18]. However, longitudinal findings suggest that fear and practice of prevention behaviors have had inverse trajectories throughout the pandemic, and therefore, are not positively associated [20]. Our findings support the latter of these studies and add to the literature suggesting that fear is not an effective predictor of prevention behavior over a span of 6 months among US women included in the sample.

### Limitations

There were a number of limitations to consider in this study. The COPE Study used an internet-based sample recruited through Facebook advertising, enabling broad reach, as most women have access to internet and use Facebook [32]; however, this resulted in variable data completion rates and quality, despite the presence of validity checks within the survey. Additionally, this is a secondary, exploratory analysis of data intended to capture women’s interpersonal experiences; assessing prevention behavior and fear of COVID-19 was not the primary focus, and therefore, the data captured are not ideal for this application. Measures used for prevention behavior were captured using a dichotomous variable, restricting the range of responses and prohibiting the exploration of more nuanced dynamics of prevention behavior frequency. A validated scale for fear of COVID-19 was used in this study, but the scale was developed rapidly in the midst COVID-19 pandemic; therefore, development may not have been as rigorous, possibly jeopardizing validity; furthermore, recent findings have documented issues with measurement invariance across countries [33]; however, reliability of the scale was strong at both time points. Participant data were only available for 2 time points, underscoring the need for caution in causal interpretation; additional time points would strengthen causal inference in future research. Maximum likelihood estimation was used in order to use all available data without listwise deletion. Upon removal of participants from the denominator who failed validity checks, an 84% (200/237) completion rate indicates that there is the small possibility of some response bias. However, validity checks were only performed at t_2_, and the high rate of failure of validity tests suggests that t_1_ data may have faced similar challenges in terms of invalid responses; however, only data from participants who passed t_2_ validity checks were included in the analysis, ensuring at least one layer of assurance and increasing the likelihood of only valid data being included. It is possible that a causal relationship exists between fear and prevention behaviors that was not identified in this study due to the length of time between assessments; we were not able to assess if fear had a more proximal but not cross-sectional impact on prevention behaviors. Finally, although the average age and racial or ethnic distribution of participants is similar to that of the US population of women, this sample is not representative of US women and may not adequately represent women without access to the internet or regular use of social media, potentially underrepresenting low-income or older individuals who may be most at risk for COVID-19. Each of these should be taken into consideration to weigh against analysis findings.

### Conclusions

Despite these limitations, this exploratory analysis uses innovative methods to examine the directionality of an important relationship hypothesized in the public health sphere—that between fear of a health outcome and prevention behaviors related to the outcome—in the highly relevant context of COVID-19. These findings have implications for public health educational and communication efforts; particularly, it may not be effective to emphasize fear as a public health tactic to promote COVID-19 prevention behaviors. The ethicality of fear appeals, particularly given the potential lack of effectiveness, should continue to be discussed; this is particularly true under circumstances of identified effectiveness, wherein there should be sustained conversation of what is effective versus what is acceptable and appropriate. Formative research on the effectiveness of fear-provoking public health campaigns should be rigorously conducted to ensure cost-effective distribution of funding to effective educational and behavior change campaigns. Further, practitioners and researchers may want to consider the nuanced dynamics of fear, when fear may not be equally applied to others (particularly those who are immunocompromised or otherwise at risk) and oneself, limiting its influence on personal behaviors. Alternative methods, such as changing negative attitudes about prevention behaviors and improving subjective norms [34], should be explored for their feasibility and effectiveness in altering prevention behaviors in the context of COVID-19. Enlisting opinion leaders within communities disproportionately affected by COVID-19 is critical in this effort, as opinion leaders can act as gatekeepers for prevention efforts, help change social norms, and accelerate behavior change [35]. Simultaneously, community outreach and education programs can be used to maximize uptake and adherence to prevention behaviors.

## Abbreviations

t_1_: time 1
t_2_: time 2
